# Diagnostic value of serologic indexes combined with Gleason score in bone metastases of prostate cancer

**DOI:** 10.1097/MD.0000000000049298

**Published:** 2026-06-12

**Authors:** Yuexi Kang, Xin Luo, Qinping Zeng, Renfei Zhang, Hao Dang

**Affiliations:** a Department of Clinical Laboratory, The Third Hospital of Mianyang, Sichuan Mental Health Center, Mianyang, Sichuan, China; b Department of Echocardiography, The Third Hospital of Mianyang, Sichuan Mental Health Center, Mianyang, Sichuan, China.

**Keywords:** bone metastasis, combined assay, fibrinogen, Gleason score, prostate cancer

## Abstract

This study aimed to explore the diagnostic value of combining serologic indexes with the Gleason score (GS) in identifying prostate cancer bone metastasis. We included 108 patients diagnosed with prostate cancer at the Third Hospital of Mianyang from December 2019 to December 2021. Clinical data were collected, and patients were divided into 2 groups: 44 cases with prostate cancer bone metastasis and 64 cases without bone metastasis. We compared age, serum total prostate-specific antigen (TPSA), free prostate-specific antigen (FPSA), fibrinogen (FIB), alkaline phosphatase (ALP), serum albumin (ALB), and GS between the 2 groups. Logistic regression analysis was used to assess the correlation between serologic indexes and prostate cancer bone metastasis. The diagnostic value of combining multiple indexes versus single indexes was evaluated using receiver operating characteristic curves. The serum TPSA, FPSA, FIB, ALP and GS levels of patients in the prostate cancer bone metastasis group were higher than those of patients in the prostate cancer without bone metastasis group, while the ALB level in the bone metastasis group was lower than that in the non-bone metastasis group. The differences in each index were statistically significant when compared between the 2 groups (*P* < .05). The univariate logistic analysis revealed that TPSA (*OR*:0.984, *P* < .001), FPSA (*OR*:0.899, *P* < .001), ALP (*OR*:0.986, *P* < .05), and GS (*OR*:0.569, *P* < .05) were positively correlated with prostate cancer bone metastasis. Multivariate analysis showed that ALP (*OR* = 0.991, *P* < .05) was an independent risk factor for bone metastasis in prostate cancer after adjusting for Age, ALB, and FIB. The area under the receiver operating characteristic curve (AUC) of serum ALP and the combined assay were 0.738 and 0.792, respectively. Serum TPSA, FPSA, FIB, ALB, ALP and GS have significant diagnostic value in identifying prostate cancer bone metastasis. ALP is an independent risk factor for bone metastasis in prostate cancer. Combining these indicators can enhance diagnostic efficacy.

## 1. Introduction

As one of the most common malignancies in elderly men, the incidence of prostate cancer (Pca) has been on the rise in recent years. According to relevant statistics, more than 2 million men worldwide suffer from prostate cancer every year, of whom more than 740,000 die of prostate cancer.^[1]^ Bone metastases are the most common cause of death in patients with Pca. For those with bone metastases, treatment becomes significantly more challenging and costly, and overall mortality rates are markedly elevated. Currently, whole-body bone scanning is routinely used in clinical practice to screen for the occurrence of bone metastases in tumors. However, the technique has poor specificity. It is costly and increases the risk of radiation exposure to patients.^[2]^ Therefore, it is crucial to identify potential predictors to better assess the severity of Pca more accurately.

Prostate-specific antigen (PSA) is an important marker for prostate cancer screening with high tissue specificity. However, elevated PSA levels are also observed in many benign prostate diseases, leading to high rates of underdiagnosis and misdiagnosis.^[3]^ The Gleason score (GS) is the most widely used pathological grading system for prostate cancer.^[4]^ A GS > 5 is considered an unfavorable factor for cancer progression in patients with prostate cancer. Fibrinogen (FIB) is an acute phase protein produced mainly by the liver. Its levels increase during malignancy and inflammation. Moreover, FIB has been closely associated with the poor prognosis of some malignancies, such as respiratory, digestive, and urinary cancers.^[5,6]^ Albumin (ALB) is generally decreased during the development of malignant tumors. It can indicate the nutritional status of the whole body at an early stage.^[7–9]^ Alkaline phosphatase (ALP) is one of the earliest biochemical indicators used in Pca testing.^[10]^ ALP is present in almost all tissues of the body but is particularly abundant in the liver and bone (osteoblasts). Studies have shown ALP is mainly secreted by osteoblasts, and its secretion increases when bone metastasis occurs in malignant tumors.^[11,12]^ Elevated serum ALP activity can thus serve as a diagnostic indicator of bone disease.^[13]^

Several researchers have suggested^[14]^ that the tumor microenvironment plays a crucial role in the occurrence and progression of malignant tumors. This microenvironment encompasses various factors, including coagulation and hemostasis, nutrient metabolism, and immunoinflammatory responses. Given the multifaceted nature of the tumor microenvironment, the present study aims to investigate the association between serological indices combined with GS and bone metastasis in prostate cancer (PCa). Additionally, this study explores the clinical application value of multi-indicator combined detection in patients with prostate cancer bone metastasis.

## 2. Materials and methods

### 2.1. Patient enrollment

In this study, 108 patients diagnosed with prostate cancer at The Third Hospital of Mianyang from December 2019 to December 2021 were included. The subjects were divided into 2 groups: the prostate cancer bone metastasis group (n = 44 cases) and the prostate cancer without bone metastasis group (n = 64 cases).

Inclusion Criteria were as follows: patients met the clinical diagnostic criteria for prostate cancer and were diagnosed by pathology; complete clinical data were available; bone metastasis of prostate cancer was diagnosed by bone scanning.

Exclusion criteria included: patients who had received radiotherapy or chemotherapy prior to surgery; patients with a history of liver disease; patients with hematological diseases, autoimmune diseases, bone injury, or other malignancies; patients whose peripheral clotting indexes were affected by anticoagulant drugs taken in the last 2 weeks; patients with incomplete clinical data.

### 2.2. Clinical date and measurement of index

The general data of all patients were collected, including age, Gleason score (GS), total prostate-specific antigen (TPSA), free PSA (FPSA), alkaline phosphatase (ALP), fibrinogen (FIB), and albumin (ALB). TPSA and FPSA were detected by chemiluminescence; ALP was detected by NPP substrate-AMP buffer assay; FIB was detected by coagulation assay; and ALB was detected by bromocresol green assay. GSs were graded by referring to the Gleason grading system, and were classified into 5 grades according to the degree of differentiation of the cells in descending order: Grade 1: GS ≤ 6 points; Level 2: GS 3 + 4 = 7 points; Level 3: GS 4 + 3 = 7 points; Level 4: GS 4 + 3, 3 + 5, 5 + 3 = 8 points; Level 5: GS 9 to 10 points.

### 2.3. Statistical analysis

SPSS 26.0 (Chicago) software was used for statistical analysis. The median (interquartile spacing) [*M(P25-P75*)] was used to express the measurement data that did not conform to a normal distribution. The Mann–Whitney *U* test was used to analyze the non-normally distributed variables. Logistic regression analysis was used to analyze the factors affecting bone metastasis of prostate cancer, and the receiver operating characteristic (ROC) curve was employed to determine the predictive value of the variables. All *P* < .05 were indicated to be statistically significant.

### 2.4. Compliance with ethical standards

This study was reviewed and approved by the Third Hospital of Mianyang (Sichuan mental health center), China.

## 3. Results

### 3.1. Comparison of general information and laboratory parameters between the 2 groups

When comparing the prostate cancer bone metastasis group with the group without bone metastasis, the difference in age between the 2 groups was not statistically significant (*P* > .05); while the differences in TPSA, FPSA, ALP, FIB and GSs were statistically significant between the 2 groups (*P* < .05). See Table 1.

**Table 1:** Comparison of general information and laboratory index levels between 2 groups.

Variables	Bone metastasis group (n = 44)	None- bone metastasis group (n = 64)	*Z* value	*P*-value
Age(in years)	75.50 (69.00, 80.00)	75.00 (68.25, 80.00)	−0.072	.943
TPSA(ng/mL)	147.00 (52.95, 154.00)	24.98 (12.58, 93.47)	−4.071	.000
FPSA(ng/mL)	19.80 (3.93, 20.10)	2.66 (1.22, 7.91)	−3.859	.000
ALB(g/L)	39.90 (36.83, 42.88)	41.65 (39.33, 44.75)	−2.020	.043
ALP(U/L)	106.50 (76.00, 208.25)	80.00 (68.00, 91.75)	−4.187	.000
FIB(g/L)	3.86 (3.19, 4.50)	3.35 (2.71, 4.31)	−2.335	.020
Gleason score				
≤7points	3 (7%)	40 (62.5%)	−3.007	.003
>7points	41 (93%)	24 (37.5%)		

ALB = serum albumin, ALP = alkaline phosphatase, FIB = fibrinogen, FPSA = free prostate-specific antigen, GS = Gleason score, TPSA = total prostate-specific antigen.

### 3.2. Analysis of factors influencing the occurrence of bone metastasis of prostate cancer

Univariate analysis demonstrated that TPSA (OR:0.984, *P* < .001), FPSA (OR:0.899, *P* < .001), ALP (OR:0.986, *P* = .003) and GS (OR:0.569, *P* = .005) were significantly correlated with bone metastasis of Pca. Multivariate analysis showed that ALP (OR:0.991, *P* = .040) was an independent risk factor for bone metastasis of Pca after adjusting for Age, ALB, FIB (Table 2).

**Table 2:** Results of univariate and multivariate logistic regression analysis.

Variables	Univariate mode OR (95% CI)	*P*-value	Multivariate mode OR (95% CI)	*P*-value
Age(in years)	1.000 (0.953–1.049)	.992	–	–
TPSA(ng/mL)	0.984 (0.978–0.991)	.000	0.983 (0.966–1.001)	.065
FPSA(ng/mL)	0.899 (0.854–0.945)	.000	1.045 (0.916–1.193)	.511
ALB(g/L)	1.096 (0.998–1.205)	.056	–	–
ALP(U/L)	0.986 (0.977–0.995)	.003	0.991 (0.982–1.000)	.040
FIB(g/L)	0.694 (0.462–1.042)	.078	–	–
Gleason score	0.569 (0.385–0.841)	.005	0.684 (0.425–1.101)	.118

ALB = serum albumin, ALP = alkaline phosphatase, CI = confidence interval, FIB = fibrinogen, FPSA = free prostate-specific antigen, GS = Gleason score, OR = odds ratio, TPSA = total prostate-specific antigen.

### 3.3. Diagnostic value of ALP and its combination of multiple indexes in prostate cancer bone metastasis

**Figure 1.**
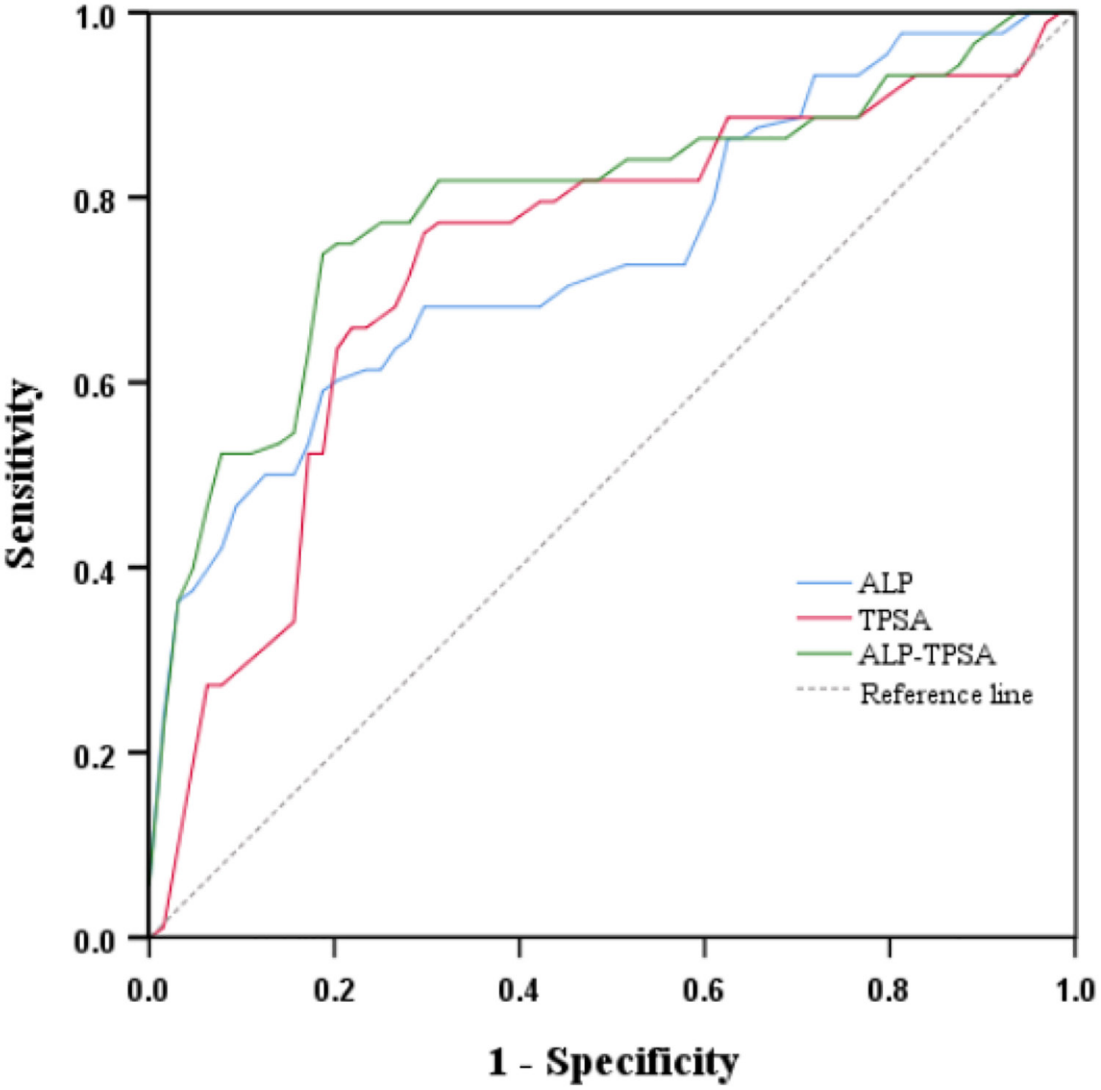
ROC curve of ALP and combined parameters. ALP = alkaline phosphatase, ROC = receiver operating characteristic.

The AUC of diagnosing prostate cancer bone metastasis with ALP level alone was 0.738 (95% *CI*:0.639~0.836), with sensitivity 61.4% and specificity 79.7%. When using TPSA for prediction, the AUC was 0.731 (95% *CI*: 0.630~6300.831), while the sensitivity and specificity were 77.3% and 70.3%. ALP and TPSA were combined to predict bone metastasis. The results showed an AUC of 0.792 (95% *CI*:0.699~0.885), with sensitivity and specificity rates of 75.0% and 81.3%, respectively (Table 3 and Fig. 1).

**Table 3:** Detailed parameters of ROC curve.

Variables	Youden index	Cut off	Sensitivity	Specificity	Area under curve	*P*-value (95% CI)
ALP (U/L)	0.411	94	0.614	0.797	0.738	<.001 (0.639–0.836)
TPSA(ng/mL)	0.476	51.27	0.773	0.703	0.731	<.001 (0.630–0.831)
TPSA-ALP	0.563	-	0.750	0.813	0.792	<.001 (0.699–0.885)

ALP = alkaline phosphatase, CI = confidence interval, ROC = receiver operating characteristic, TPSA = total prostate-specific antigen.

## 4. Discussion

In recent years, prostate cancer has become one of the most prevalent malignant tumors among elderly men in China. According to a related study,^[15]^ approximately 3% of patients initially diagnosed with prostate cancer present with bone metastasis, and the risk of bone metastasis in these patients was found to be as high as 11.5% after 2 years of follow-up.^[16]^ Currently, the diagnosis of bone metastasis in prostate cancer relies on whole-body bone scanning. However, this technique has poor specificity, high cost, and increased radiation exposure to patients.^[17]^

In recent years, there has been a trend towards a younger population with prostate cancer. Additionally, the malignancy of prostate cancer is generally higher in patients aged > 75 years compared to those aged 55 to 75 years.^[15]^ Exploring the research reports in recent years, the value of age in prostate cancer bone metastasis is somewhat controversial. Wang et al^[18]^ found no correlation between age and the occurrence of bone metastasis in prostate cancer. In contrast, Chen^[19]^ et al concluded that age is related to prostate cancer bone metastasis but is not an independent risk factor. However, Merdan et al^[20]^ concluded that age is an independent risk factor for bone metastasis in prostate cancer. In this study, there was no certain correlation between age and the occurrence of bone metastasis of prostate cancer, which is consistent with the study of Wang et al.^[18]^ The age distribution of the study population was predominantly around 75 years, which may introduce some representational bias. Therefore, future studies should consider increasing the sample size to further explore the relationship between age and the occurrence of prostate cancer bone metastasis.

TPSA and FPSA are commonly used screening indicators for prostate diseases. When prostate cancer occurs, the proliferation and infiltration of cancer cells lead to the loss of the basement membrane of prostate follicles and ducts, resulting in a large number of PSA proteins flowing into the blood circulation through the lymphatic system or leaking into the cellular interstitial space, and then entering into the peripheral circulatory system via capillaries, which leads to the elevation of PSA in the peripheral blood.^[21]^ Elevated PSA levels have been reported in metastatic prostate cancer tissues.^[22]^ Our study found that serum PSA levels were higher in prostate cancer patients with bone metastasis than in those without. The optimal TPSA cutoff value for predicting bone metastasis remains controversial. In our study, a TPSA level > 51.27 ng/ml achieved a sensitivity of 77.3% and specificity of 70.3% for diagnosing bone metastasis, with an area under the ROC curve (AUC) of 0.731. When PSA diagnosis is uncertain, combining it with other indicators can improve diagnostic accuracy.

When there is uncertainty in the diagnosis of PSA, it can be combined with other indicators to clarify the diagnosis of prostate cancer as well as bone metastases. Relevant studies have shown that the nutritional status of patients is related to the occurrence and progression of tumors,^[23]^ and the organism of tumor patients is often accompanied by the activation of the coagulation system.^[24]^ Thus, our study evaluated the differences in the concentration levels of ALB and FIB between the 2 groups, as seen in Table 1. However, multifactorial logistic regression analysis showed that the correlation between ALB and FIB and the occurrence of prostate cancer bone metastasis was not significant (*P* > .05), which may be related to the fact that this index is susceptible to systemic inflammation and drugs, and the stability of this index is relatively weak.

Alkaline phosphatase (ALP) is widely distributed in human bones, liver, kidney, and intestinal mucosal tissues.^[25]^ Wei^[26]^ et al reported an ALP cutoff value of 115 U/L for diagnosing prostate cancer bone metastasis, with a sensitivity of 57.1% and specificity of 64.8%. In our study, ALP levels were significantly higher in the bone metastasis group. Logistic regression analysis identified ALP as an independent predictor of bone metastasis. The ROC curve analysis showed an optimal ALP cutoff value of 94 U/L, with an AUC of 0.738, sensitivity of 61.4%, and specificity of 79.7%, consistent with previous studies.

Gleason score (GS) is widely used for prostate cancer grading. According to several studies,^[27]^ GS score is closely related to the occurrence of bone metastasis in prostate cancer, and the incidence of bone metastasis in prostate cancer patients is higher when the GS score is above 8. Our results indicated that the bone metastasis group had higher GS scores (*P* < .05), but logistic regression analysis showed that the GS score was not an independent predictor of bone metastasis in prostate cancer. This was inconsistent with the study of Chao^[28]^ and others,^[29]^ which might be related to the sample source and sample size.

The results of ROC curve analysis of single-indicator and multiple-indicator combined tests for diagnosing bone metastasis of prostate cancer showed that the efficacy of combined ALP and TPSA for diagnosing bone metastasis of prostate cancer was better than a single-indicator test, and the combined indicator had higher sensitivity and specificity. This highlights the diagnostic value of combined ALP and TPSA testing in prostate cancer bone metastasis.

In addition, TPSA, FPSA, ALB, ALP, FIB, and GS have certain diagnostic value in prostate cancer bone metastasis. ALP may be an independent predictor, and combined ALP and TPSA testing offers a valuable diagnostic approach.

## 5. Limitations

This study has several limitations, including its small sample size, single-center design, and unvalidated model incorporating ALP, TPSA, and GS. Future research should involve multicenter validation with a larger sample size to confirm these findings and enhance the robustness of the predictive model.

## 6. Conclusion

The incidence of prostate cancer with bone metastasis is significantly associated with serum TPSA, FPSA, FIB, ALB, ALP, and GS. Patients with bone metastasis exhibit elevated levels of TPSA, FPSA, ALP, FIB, and GS, while ALB levels are reduced. Moreover, these indicators are strongly correlated, and combined detection offers higher predictive value than single-indicator testing. Integrating these factors can lead to more accurate and timely diagnoses, thereby improving patient outcomes.

## Acknowledgments

We would like to thank the staff of Department of Clinical Laboratory of the Third Hospital of Mianyang for their contribution.

## Author contributions

**Data curation:** Yuexi Kang, Xin Luo.

**Investigation:** Xin Luo, Qinping Zeng.

**Project administration:** Yuexi Kang.

**Resources:** Xin Luo.

**Software:** Qinping Zeng.

**Supervision:** Renfei Zhang, Hao Dang.

**Visualization:** Renfei Zhang, Hao Dang.

**Writing – original draft:** Yuexi Kang.

**Writing – review & editing:** Yuexi Kang.
